# Androgen receptor positive triple negative breast cancer: Clinicopathologic, prognostic, and predictive features

**DOI:** 10.1371/journal.pone.0197827

**Published:** 2018-06-08

**Authors:** Kristine Astvatsaturyan, Yong Yue, Ann E. Walts, Shikha Bose

**Affiliations:** 1 Department of Pathology and Laboratory Medicine, Cedars-Sinai Medical Center, Los Angeles, California, United States of America; 2 Department of Radiation Oncology, Cedars-Sinai Medical Center, Los Angeles, California, United States of America; University of South Alabama Mitchell Cancer Institute, UNITED STATES

## Abstract

**Introduction:**

Overexpression of the androgen receptor (AR) characterizes a distinct molecular subset of triple negative breast carcinomas (TNBC). The role of AR as a prognostic/predictive biomarker in TNBC is controversial, but increasing evidence suggests that this subset may respond to therapeutic agents targeting AR. Evaluation of AR has not been standardized, and criteria for selection of patients for antiandrogen therapy remain controversial. In this study we determine the appropriate threshold of AR immunoreactivity to define AR positive (AR+) TNBC, describe the clinicopathologic features of AR+ TNBC, and discuss the utility of AR positivity as a prognostic and predictive marker in TNBC.

**Materials and methods:**

135 invasive TNBC processed in accordance with ASCO/CAP guidelines, were immunostained for AR. Clinicopathologic features of AR+ TNBC were analyzed and compared to AR negative (AR-) TNBC. Patients’ age, tumor size, tumor grade, lymph node status, proliferation rate, immunopositivity for EGFR, CK5/6, Ki-67, and disease free survival (DFS) were evaluated statistically.

**Results:**

A 1% cutpoint was confirmed as the appropriate threshold for AR positivity. Using this cutpoint 41% of 135 TNBC were AR+. AR+ TNBC occurred in older women, were larger, had lower mean proliferation rate and increased incidence of axillary metastasis than AR- TNBC. 76% of TNBC with apocrine morphology were AR+. A subset of AR+TNBC expressed basal markers (EGFR and CK5/6). A prognostic model was created.

**Summary:**

AR identifies a heterogeneous group of TNBC. Additional evaluation of EGFR expression allowed us to stratify TNBCs into 3 risk groups with significant differences in DFS and therapeutic implications: low-risk (AR+ EGFR-) which represents the LAR molecular subtype with the best prognosis and may benefit the most from anti-androgen therapies; high-risk (AR- EGFR+) which represents the basal molecular subtype with the worst prognosis and may benefit the most from chemotherapy regimens; intermediate-risk (AR+EGFR+ and AR-EGFR-) TNBC with an intermediate prognosis. Prospective trials are required to further validate this prognostic and predictive grouping.

## Introduction

Triple negative breast carcinomas (TNBC) are defined by absence of expression for estrogen receptor (ER) and progesterone receptor (PR) by immunohistochemistry (IHC), absence of overexpression for human epidermal growth factor receptor HER2/neu (HER2) by IHC, and absence of amplification of HER2 by fluorescent in situ hybridization (FISH). TNBC accounts for 10–20% of newly diagnosed breast cancers [1]. They tend to be larger and higher grade, have a higher incidence of axillary lymph node metastasis and are associated with worse overall survival than other types of breast carcinoma [2–9].

Molecular analyses have shown that TNBC is a heterogeneous disease. As reported by Lehmann et al., TNBC can be further classified into four molecular subtypes [basal-like1, basal-like2, mesenchymal, and luminal androgen receptor (LAR)], each characterized by different clinicopathologic features and different driver signaling pharmacologically targetable pathways [10]. In another study, Jezequel et al. demonstrated three molecular subtypes (basal with low immune response, basal with high immune response, and LAR) [11]. Subsequently, other groups have confirmed LAR as a distinct subtype of TNBC characterized by high AR expression and enrichment of hormonally regulated pathways that are important in steroid synthesis, porphyrin metabolism, and androgen/estrogen metabolism despite absence of ER [12,13].

Significant variability exists in the reported literature regarding the frequency of AR expression in TNBC with values ranging from 7–75% [14–19]. The role of AR expression as a prognostic factor in TNBC is also not clear. It has been reported as a favorable prognostic factor (associated with low grade, low stage, low proliferative rate tumors) [7, 19–24], as an unfavorable prognostic factor (associated with increased lymph node metastasis, increased mortality, and poor disease free survival) [25–27], and as unrelated to prognosis [28–30]. Several clinical trials targeting AR expressing TNBC are ongoing and have shown promise; however, considerable variability exists in patient selection criteria and reported response rates are generally low [31, 32].

This study was designed to determine the threshold of AR for immuohistochemical evaluation (minimum staining required for a tumor to be considered AR+), to analyze the clinicopathologic features of TNBCs expressing AR, and to determine the utility of AR immunoexpression as a prognostic and predictive marker in TNBC. AR expression was also integrated into a prognostic model that stratified TNBC into risk groups that may be used to help guide personalized therapy.

## Material and methods

### Patients

The study protocol was approved by the Institutional Review Board of Cedars-Sinai Medical Center. The committee waived the need for informed consent. 192 consecutive invasive TNBCs diagnosed from 2008 to 2012 were retrieved from our database. TNBC were defined by lack (<1% positivity) of ER and PR immunoreactivity and a HER2/neu score of 0 or 1+ by immunohistochemistry (IHC) and absence of amplification by FISH. Clinical parameters (age, sex, tumor location, type of surgery, adjuvant chemotherapy) and pathologic features (tumor size, grade, proliferation rate, lympho-vascular invasion, axillary lymph node status, results of Ki-67, p53, EGFR and CK5/6 immunostains) were recorded. All tissue samples were fixed in 10% buffered formalin and embedded in paraffin wax for routine histological examination in accordance with the 2007 American Society of Clinical Oncology/College of American Pathologists (ASCO/CAP) guidelines [33]. Hematoxylin and eosin (H&E) stained sections of all cases were reviewed, the diagnosis of invasive carcinoma was confirmed, and a representative block was selected for AR immunostain. 135 cases contained sufficient tumor; these formed the study cohort. No additional selection criteria were applied.

### Immunohistochemistry

IHC stains were performed on whole sections using a Polymer and/or SA-HRP Detection System with appropriate positive and negative controls. ER, PR, Ki-67, p53, and HER2/neu labeling indices were determined using SP1, 1E2, K-2, DO7, and 4B5 antibodies (Ventana Medical Systems, Tucson, AZ, USA), respectively. ER, PR, Ki-67 and p53 immunoexpression was evaluated as the percentage of cells exhibiting nuclear staining. Cell proliferation (Ki-67) was assessed by counting at least 500 tumor cell nuclei (depending upon the availability of tumor) and graded as low (<11%), intermediate (11–20%), and high (>21%) [34]. Immunostaining for EGFR and CK 5/6 was performed using the monoclonal antibody 2-18C9 for EGFR (Dako, Glostrup, Denmark) and D5/16 B4 for CK5/6 (Dako, Glostrup, Denmark). Results were recorded as the percentage of invasive carcinoma cells showing cytoplasmic and/or membrane staining. For EGFR, staining in >15% of tumor cells was interpreted as positive and staining in ≤15% of tumor cells was interpreted as negative. For CK5/6, staining in >50% of tumor cells was interpreted as positive and staining in ≤50% of tumor cells was interpreted as negative, as previously reported [16].

Immunohistochemical detection of Androgen Receptor was performed on 4-μm whole tissue sections with antibody clone F39.4.1 (Biogenex, Fremont, CA) applied at 1:100 dilution and incubated for 45 minutes at room temperature. Pretreatment was performed by the Dako PT Link module with low pH buffer (Carpenteria, CA). Staining was done on the Dako Autostainer and visualized by the Dako Envision mouse detection system at 30-minute incubation using Dako DAB and subsequent counterstaining with Mayer’s hematoxylin. The percentage of tumor nuclei that stained was recorded.

All cases were evaluated independently by two pathologists. Consensus results were recorded and discordances were resolved by review and discussion.

### Statistical analyses

Statistical analysis was performed using R software 3.1.1 (The R foundation for statistical computing, Vienna, Austria) and SPSS 17.0 statistical software (SPSS Inc, Chicago, IL, USA). The Wilcoxon test was used to compare the clinicopathologic characteristics of AR+ and AR- TNBCs. Survival differences between the two groups were compared using log-rank tests and Kaplan-Meier curves. Disease-free survival (DFS), defined as the interval between period after curative treatment and the date of first recurrence or progression of disease, or the date of death from any cause, was used as the endpoint for survival analysis. Patients who were not reported to be dead at the time of the analysis were censored at the date they were last known to be alive.

### Determination of the threshold for AR positivity

In order to determine the appropriate threshold for AR positivity the 135 cases were divided into 2 groups: Group A (the study set; n = 35) and Group B (the validation set; n = 100). Tumor characteristics and DFS survival were similar in the two groups. Additionally, DFS survival of EGFR+ and EGFR- cases in the two groups was also similar (S1 Fig). DFS was analyzed with AR defined as positive when 1%, 10%, 20%, 25%, and 30% of tumor nuclei stained.

### Creation of prognostic model

Prognostic factors were determined using Cox proportional hazard regression analysis. The resultant significant biomarkers/variables were selected as candidate prognostic indicators, and the prognostic contributions of a combination of biomarkers were identified by multivariate Cox analysis [31]. Patients were further stratified into different risk categories based on the expression values of the various biomarkers. The difference in DFS between the various risk categories was evaluated by log-rank tests and Kaplan-Meier curves. A p-value less than 0.05 was considered significant.

## Results

**Fig 1:**
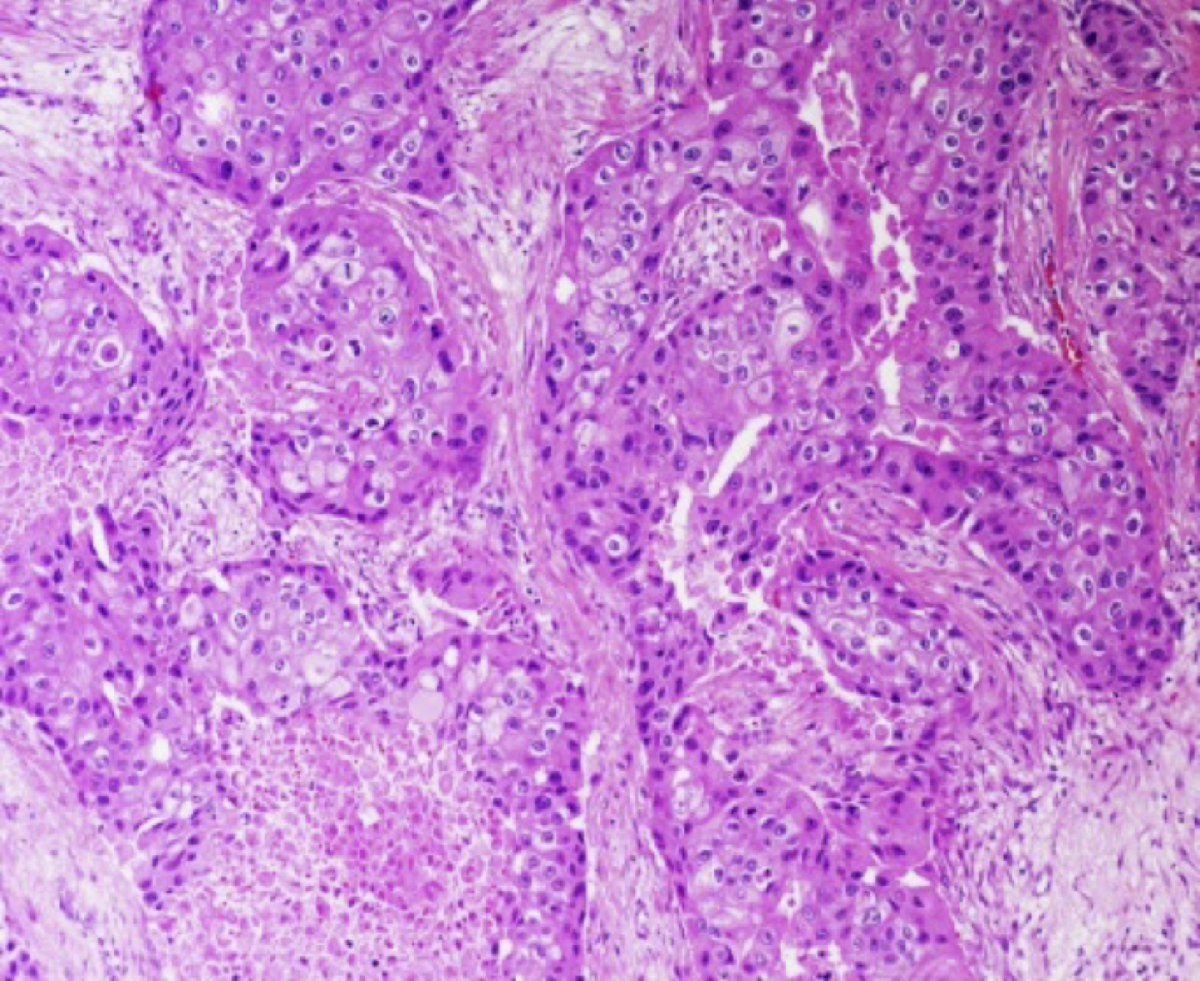
Apocrine differentiation in AR positive TNBC. Hematoxylin and Eosin (H&E) stain, 20x.

The 135 women ranged in age from 28 to 92 (median 57, mean 58) years at the time of TNBC diagnosis. Each patient had received lumpectomy or mastectomy as primary treatment followed by chemotherapy and radiation therapy. Follow-up ranged from 2 to 54 (median 22) months. Tumor size varied from 0.3 to 10.3 (median 2.4, mean 2.6) cm. 98% (132/135) of the TNBC were infiltrating ductal carcinomas of which 13% (17) showed apocrine differentiation (round nuclei with prominent nucleoli and abundant granular cytoplasm; ApoCA+) in at least 70% of the neoplastic cells (Fig 1). The remaining 3 TNBC were mixed ductal and lobular (n = 1) and metaplastic (n = 2). 90% (121/135) of the TNBC exhibited high histologic grade, 85% (115/135) had high proliferative index, and 70% (94/135) expressed EGFR, a basal marker. Only 31% (42/135) had axillary lymph node metastasis.

### Establishing a threshold to define AR positivity

**Fig 2:**
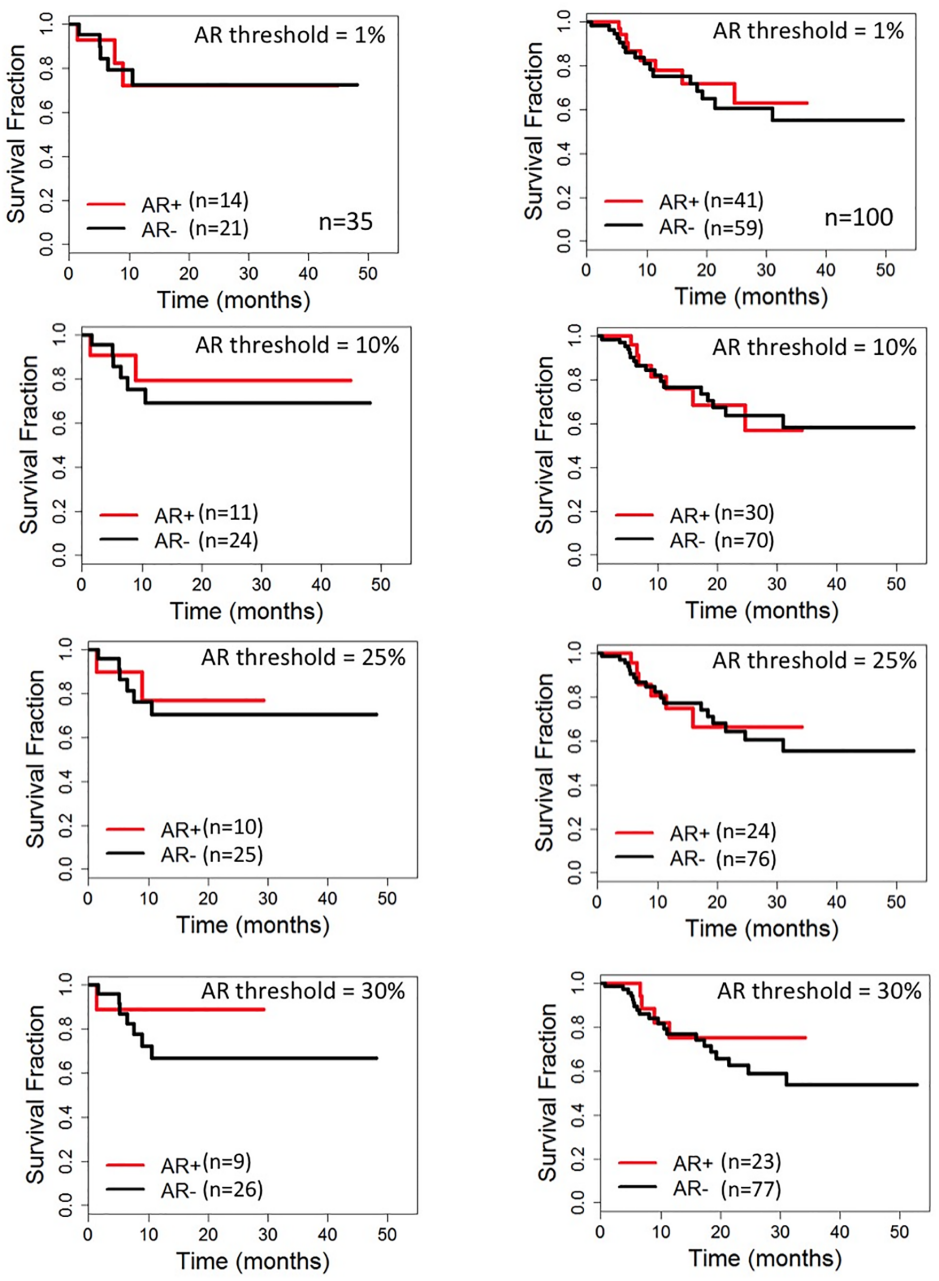
Disease free survival of patients with AR positive and AR negative TNBC. Group A, left column (study set, n = 35), and Group B, right column (validation set, n = 100) at different thresholds of 1%, 10%, 25% and 30% from top to bottom.

No significant difference was observed in DFS within Group A or within Group B when 1%, 10%, 20%, 25% or 30% staining was used as the threshold for AR positivity (Fig 2, S1A and S1B Table). Clinicopathologic features of all 135 cases were also evaluated using 1% and 25% staining as the threshold for AR positivity. At the 1% cutpoint AR positive (AR+) tumors were significantly larger and showed a higher incidence of lymph node metastasis than AR negative (AR-) tumors. At the 25% cutpoint, no significant differences in tumor characteristics were observed between AR+ and AR- tumors. Thus, AR immunoreactivity in at least 1% of tumor cell nuclei was considered the most appropriate threshold to define AR positivity.

### Clinical and pathologic features of AR+TNBC

#### Prevalence

**Fig 3:**
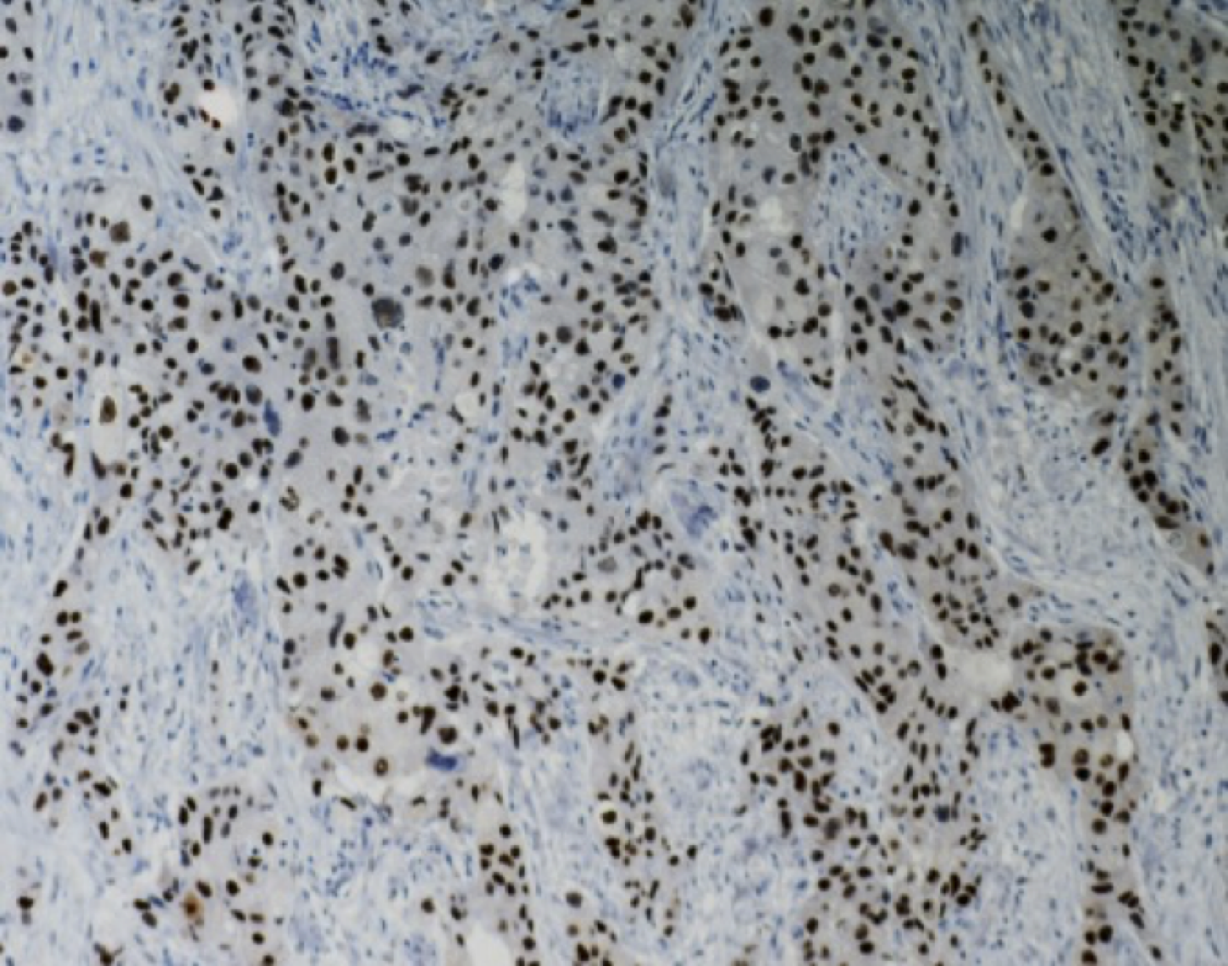
AR immunoexpression in TNBC. >50% of neoplastic cells are positive. 20x.

When 1% was utilized as the threshold to define AR positivity, 41% (55/135) of the TNBC were AR+ (Table 1). Among the AR+ tumors, staining was strong (≥50% positive, Fig 3) in 45%, intermediate (25–50% positive) in 16% and weak (1–24% positive, S2 Fig) in 39% of the 55 cases, corresponding to 18.5%, 7%, and 15%, respectively, of all 135 TNBC in the study. The remaining 80 TNBC were AR- (<1% positive).

**Table 1:** Clinicopathologic features of AR positive and AR negative TNBC.

Variables	Total (%)	AR+ (≥1%) No. of Patients (%)	AR- (<1%) No. of Patients (%)	Wilcoxon Test p-Value
**Total Number of Patients (%)**	135 (100%)	55 (41)	80 (59)	
**Age**				**0.015**
<50	42 (31)	13 (24)	29 (36)	
50–70	64 (47)	25 (45)	40 (49)	
>70	29 (21)	17 (31)	12 (15)	
Mean	57.4	61.4	54.8	
**Tumor Size**				**0.027**
≤20mm	62 (46)	20 (36)	42 (52)	
>20 and ≤50mm	61 (45)	28 (51)	33 (42)	
>50	12 (9)	7 (13)	5 (6)	
Mean	31 mm	31 mm	23 mm	
**Apocrine**				**0.001**
Yes	17 (13)	13 (24)	4 (5)	
No	118 (87)	42 (76)	76 (95)	
**MBR score**				0.842
I	1 (1)	1 (2)	0	
II	13 (9)	5 (9)	8 (10)	
III	121 (90)	49 (89)	72 (90)	
**Lymph Node Status**				**0.009**
pN0	82 (61)	28 (51)	54 (68)	
pN1 (1–3)	31 (23)	18 (33)	13 (17)	
pN2 (4–9)	9 (6.5)	3 (5)	6 (8)	
pN3 (10 and >)	2 (1.5)	1 (2)	1 (1)	
pNx	11 (8)	5 (9)	6 (7)	
**Stage**				0.469
I	39 (29)	13 (24)	26 (32)	
II	63 (46)	28 (5)	35 (43)	
III	15 (11)	7 (13)	8 (10)	
IV	5 (4)	1 (2)	4 (5)	
NA	13 (10)	6 (7)	7 (9)	
**Ki-67**				**0.014**
≤10%	6 (5)	3 (6)	3 (4)	
11–21%	14 (10)	9 (16)	5 (6)	
>21%	115 (85)	43 (78)	72 (90)	
**EGFR**				**0.023**
≤15%	41 (30)	13 (24)	28 (35)	
>15%	94 (70)	42 (76)	52 (65)	
**CK5/6**				0.086
≤50%	101 (75)	44 (80)	57 (71)	
>50%	34 (25)	11 (20)	23 (29)	
**Disease Free Survival, Mean**		35.8±2.63	34.1±2.63	0.651

AR, androgen receptor; TNBC, triple negative breast cancer; MBR, modified Bloom-Richardson; EGFR, epidermal growth factor receptor; CK, cytokeratin.

#### Age of patients

Women with AR+TNBC ranged from 29 to 89 years. The mean age of women in the AR+ group was significantly older than that of women in the AR- group (61.4 vs. 54.8 yrs; p = 0.015).

#### Tumor size

AR+TNBC varied in size from 0.6 to 10.3 (mean 3.1, median 2.9) cm. AR+TNBC were significantly larger than AR-TNBC (mean/median 3.1 cm/2.9 cm vs. 2.3 cm/2.0 cm; p = 0.027).

#### Histologic subtype

96% (53/55) of the AR+TNBC were ductal, one was lobular, and one was mixed ductal/lobular. 24% (13/55) of the AR+TNBC were ApoCA+ (Fig 1) compared with 5% (4/80) of the AR-TNBC (p = 0.001). Among the 17 ApoCA+, 76% (13/17) were AR+ with strong (≥50%) staining seen in 12 and weak (10%) staining seen in only one of the cases.

#### Histologic grade

89% (49/55) of the AR+TNBC had a high histologic grade (Modified Bloom Richardson score III). No difference was noted in tumor grade when compared to the AR-TNBC. However, AR+ApoCA+TNBC showed a lower grade when compared to AR+ApoCA-TNBC (p = <0.001). 98% (41/42) of AR+ApoCA-TNBC demonstrated a high histologic grade. In comparison 62% (8/13) AR+ApoCA+TNBC demonstrated a high histologic grade and 38% (5/13) demonstrated an intermediate histologic grade.

#### Proliferation rate

**Fig 4:**
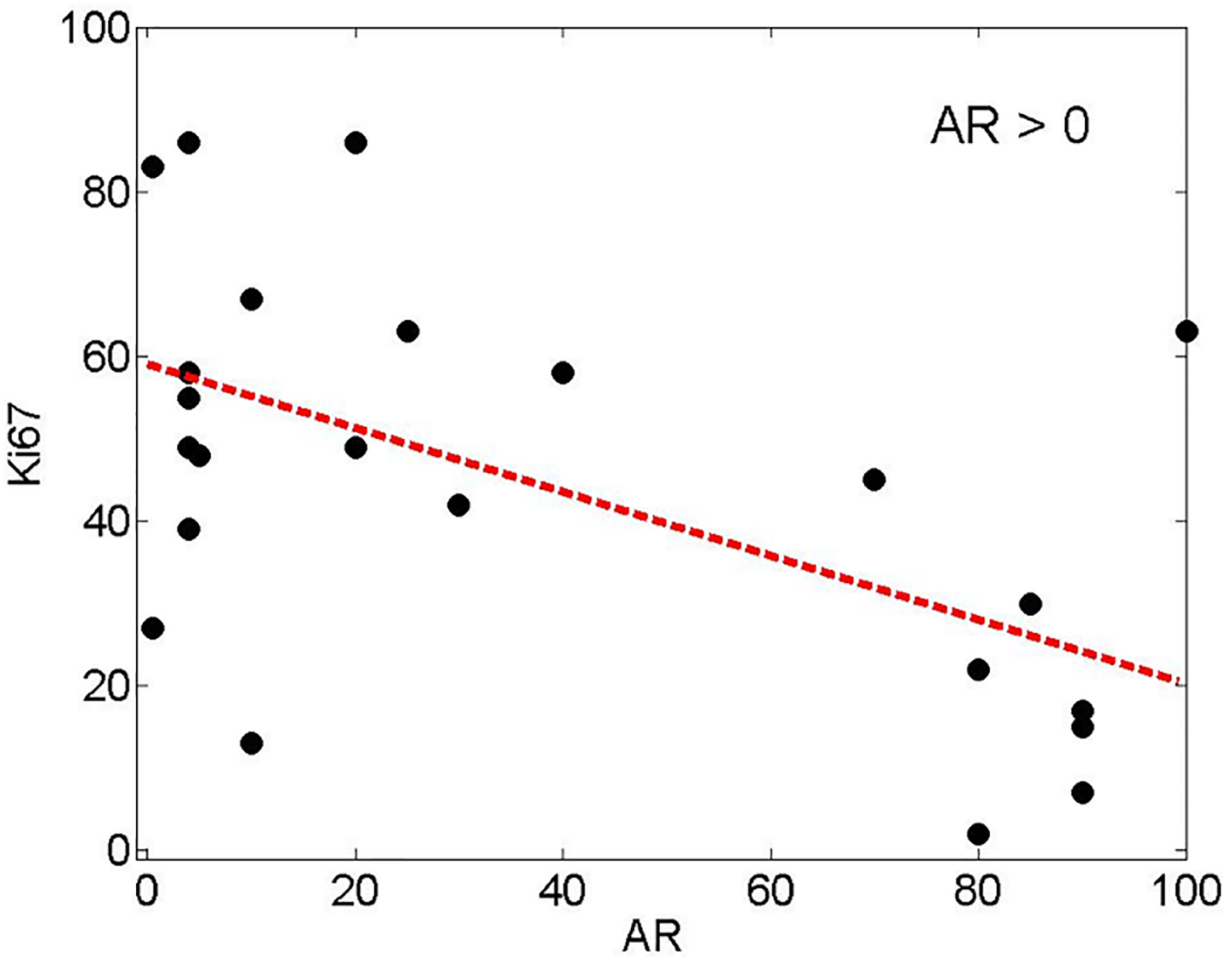
Inverse correlation of AR expression and proliferation rate as measured by Ki-67 immunostain (Correlation coefficient -0.367; p = 1.009e-5).

A statistically significant inverse correlation was observed between AR expression and proliferation rate (p = 0.014). Lower levels of AR expression were associated with higher levels of Ki-67 immunostaining (Fig 4). 78% (43/55) of AR+TNBC showed a high proliferation rate (>21%) as compared to 90% (72/80) of AR-TNBC. AR+ApoCA+ also showed a lower proliferation rate when compared to AR+ApoCA-TNBC. 31% (4/13) AR+ApoCA+TNBC showed a high proliferation rate as compared to 93% (39/42) AR+ApoCA-TNBC (p = <0.001).

#### Expression of basal markers

AR+TNBC showed variable immnoreactivity for basal biomarkers (EGFR and CK5/6). EGFR positivity was more frequently observed than CK5/6 positivity in AR+TNBC (76% vs. 20%). EGFR positivity was also more frequently seen in AR+ compared to AR- TNBC (76% VS 65%; p = 0.023). 45% (42/94) EGFR+TNBC and 23% (11/34) CK5/6+TNBC expressed AR suggesting that a subset of AR+TNBC also expresses the basal phenotype (S3 Fig).

#### Axillary lymph node metastasis

Axillary lymph node metastasis were more frequent in AR+ than AR- TNBC (40% vs. 25%; p = 0.009). This statistically significant difference was maintained when the AR+ApoCA+TNBC were compared with the AR+ApoCA- subgroup (77% vs. 29%; p = 0.004).

#### Outcome

The mean DFS in the AR+TNBC was 35.8 months. When compared to the mean DFS in the AR-TNBC (34.1 months), no significant difference was observed.

#### Summary of clinical and pathological features of AR positive TNBC

Using ≥1% AR immunoreactivity to define AR+TNBC, 41% of our study cohort were AR+. When compared to AR-TNBC, AR+TNBC were larger and more frequent in older women, showed a higher incidence of apocrine differentiation, a higher incidence of axillary lymph node metastasis, and lower proliferation rates. No siginificant difference was observed in mean DFS between the two groups. 24% exhibited prominent apocrine differentiation. AR+ApoCA+TNBC were also more frequent in older women, were larger tumors with lower proliferation rates and increased lymph node metastasis when compared to AR+ApoCA-TNBC. A subset of AR+TNBC demonstrated EGFR and CK5/6 positivity suggesting a basal phenotype.

### Prognostic model

**Fig 5:**
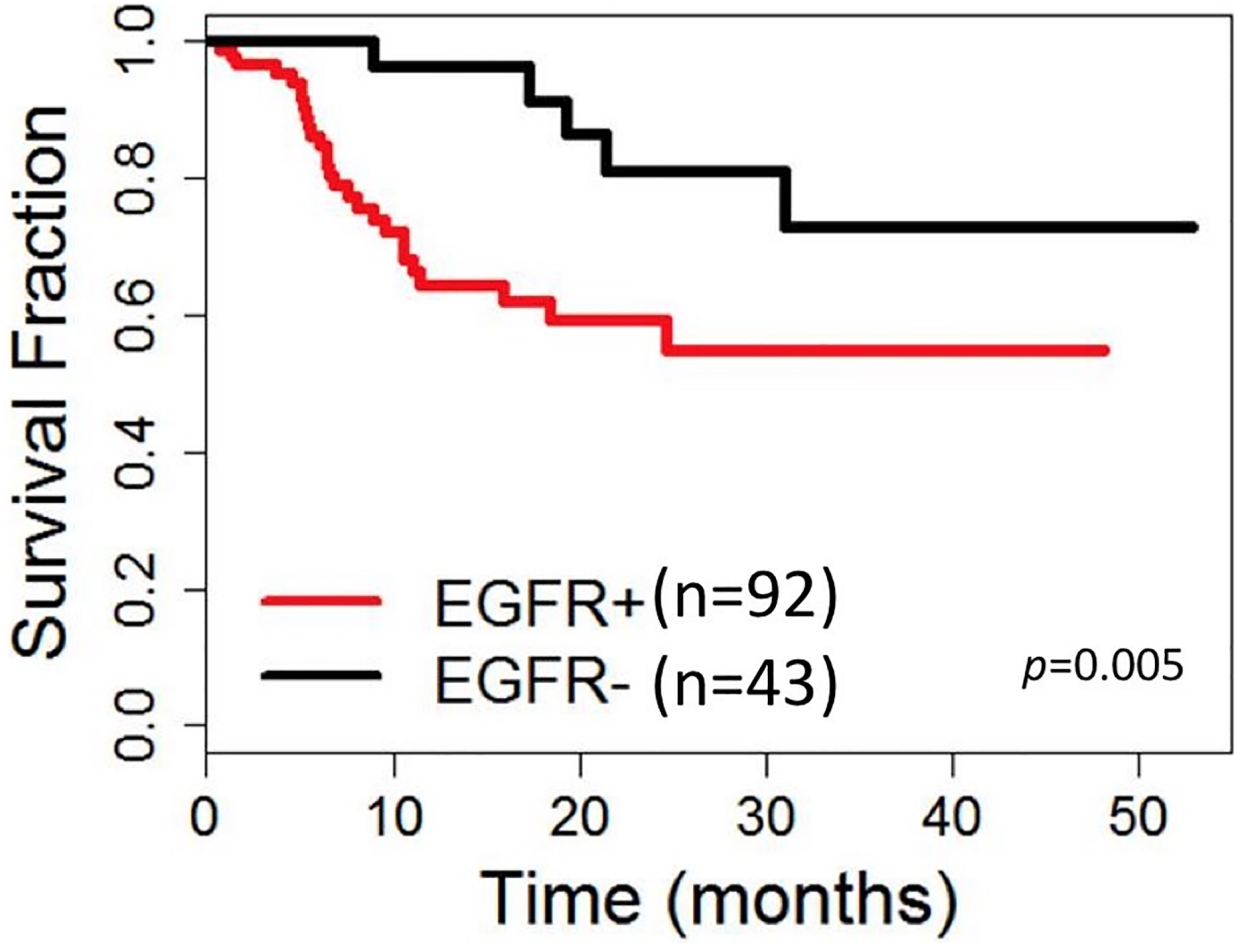
Disease free survival of EGFR positive and EGFR negative TNBC.

**Fig 6:**
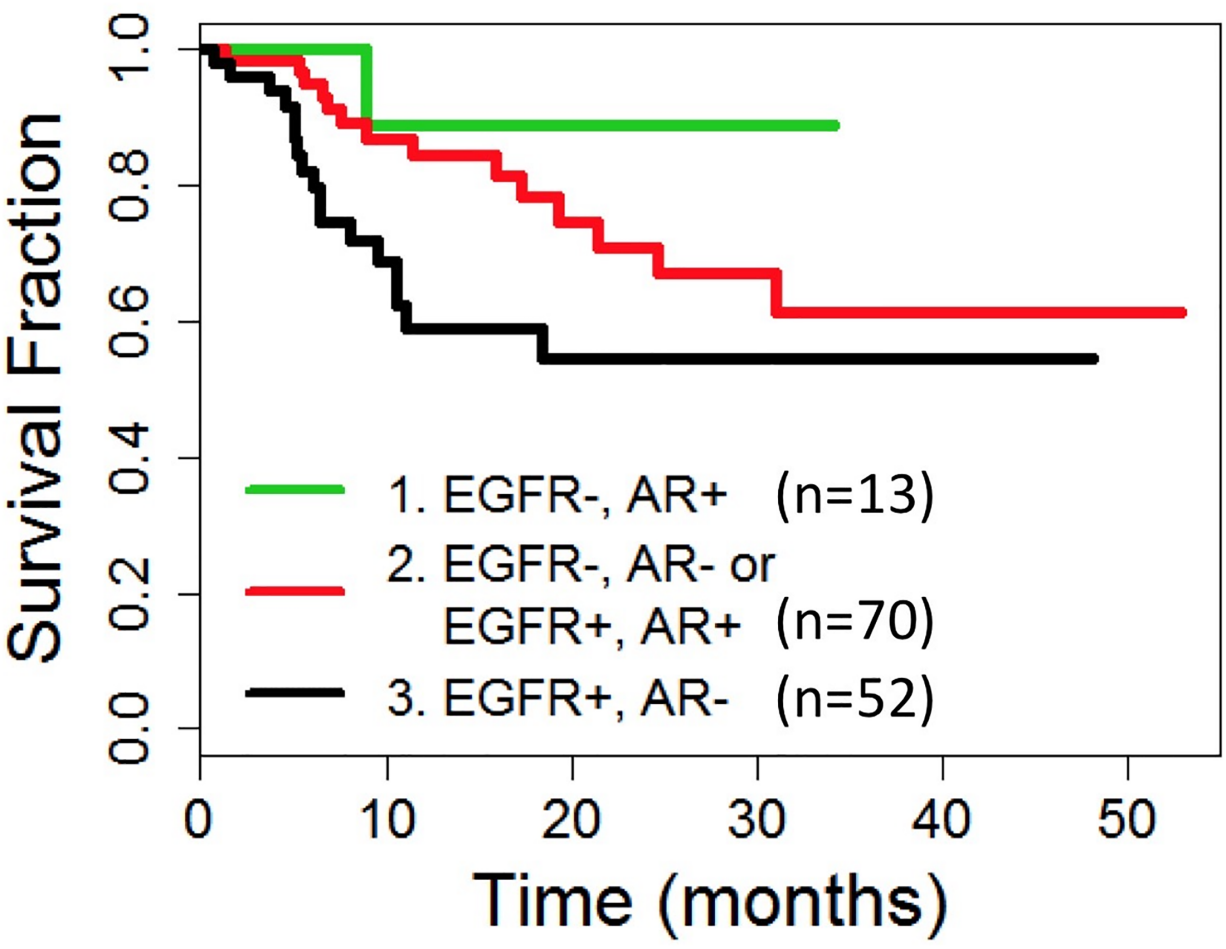
Disease free survival of TNBC stratified by AR and EGFR. Cases were stratified into three risk groups: 1. Low risk: AR+ EGFR-; 2. Intermediate risk: AR+ EGFR+ or AR- EGFR-; 3. High Risk: AR- EGFR+.

**Fig 7:**
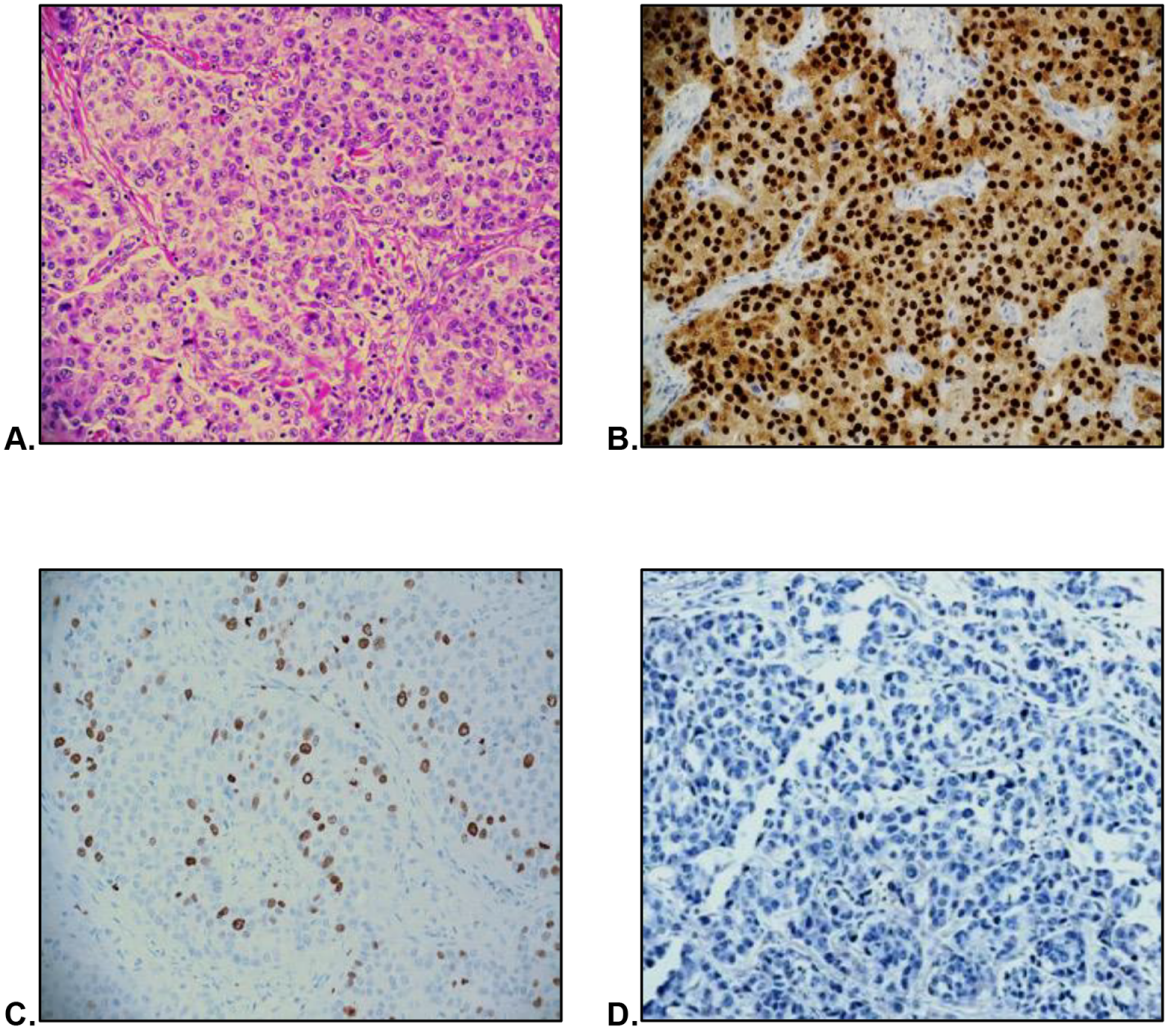
Low risk TNBC. Morphology as seen on H&E stain (A) and characterized by strong AR immunopositivity (B), low Ki-67 (C), and negative EGFR (D).

**Fig 8:**
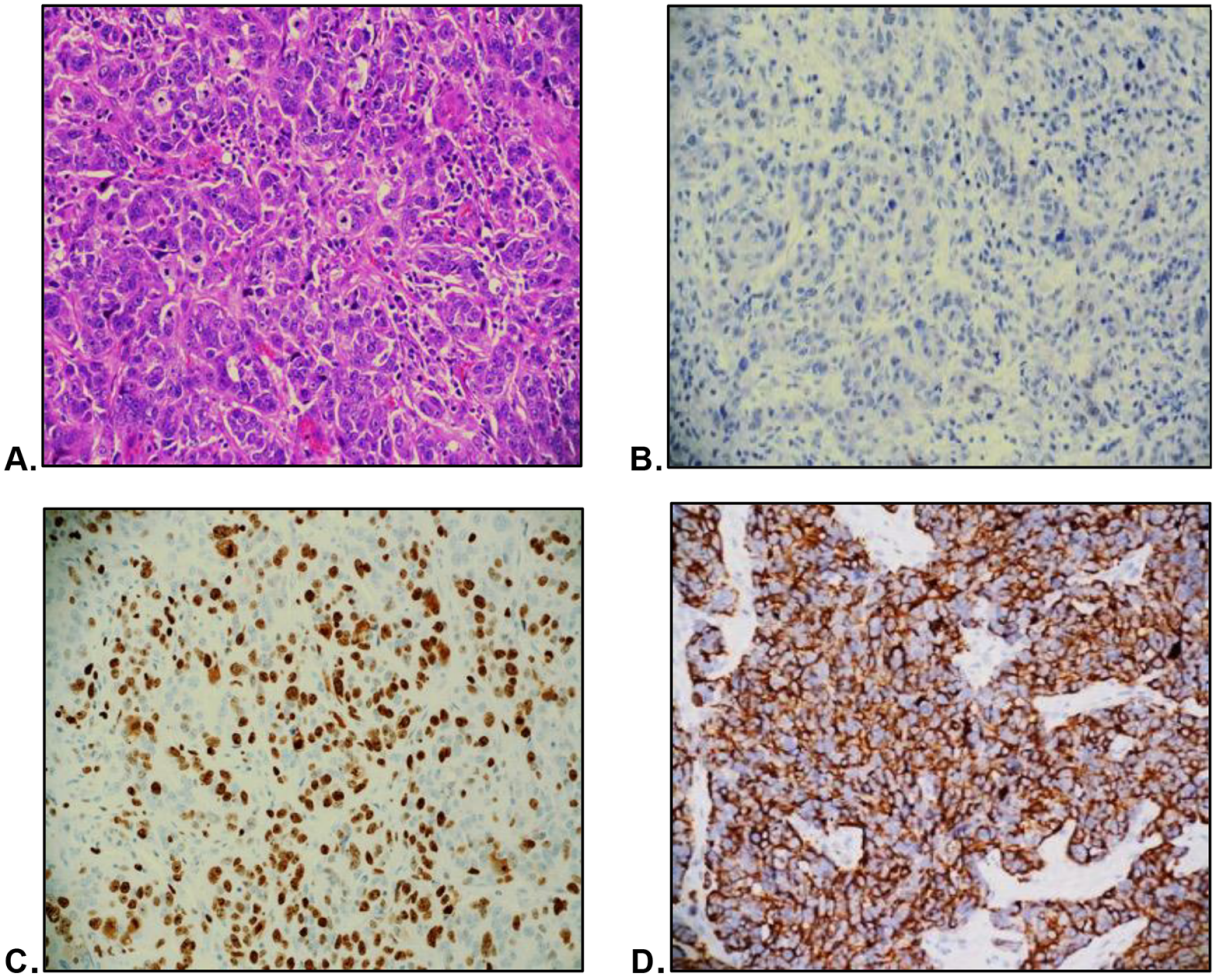
High risk TNBC. Morphology as seen on H&E stain (A) and characterized by low AR immunopositivity (B), high Ki-67 (C), and positive EGFR (D).

We previously identified EGFR immunoreactivity in ≥15% of tumor cell nuclei as a negative correlate of DFS in TNBC [16]. In the current study, EGFR+ (≥15%) TNBC also experienced a lower DFS than the EGFR- (<15%) TNBC (mean DFS 31.7 vs 43.8 months; p = 0.005, Fig 5). Although AR+TNBC did not demonstrate any survival benefit, we were able to develop a statistically significant (p = 0.0374) prognostic model using a combination of EGFR and AR which was successful in stratifying TNBC into the following three prognostic groups (Table 2, Figs 6, 7 and 8):

Group 1 (low-risk TNBC): AR+EGFR- tumors. This group had the best outcome with mean DFS of 43.7 months and only a single event (either disease recurrence or metastasis at one site).
Group 2 (intermediate-risk TNBC): AR+EGFR+ and AR-EGFR- tumors. This group had a mean DFS of 35.9 months. 19% patients in this group had ≥1 events (disease recurrence and/or metastases at multiple sites).
Group 3 (high-risk TNBC): AR-EGFR+ tumors. This group had a mean DFS of 29.8 months and the highest event rate with ≥1 events having occurred in 34% of patients. We also noted that the mean proliferation rate (Ki-67%) increased from 30% in the low-risk group to 57% in the high-risk group.

**Table 2:** Prognostic groups of TNBC as stratified by AR and EGFR.

Prognostic Groups	Patient Number (%)	AR	EGFR	Mean DFS Months	Events in the Group (%)
1. Low risk	13 (9)	Positive	Negative	43.7	1 (7)
2. Intermediate risk	70 (52)	Positive/Negative	Positive/Negative	35.9	14 (19)
3. High risk	52 (39)	Negative	Positive	29.8	17 (34)

AR, androgen receptor; TNBC, triple negative breast cancer; EGFR, epidermal growth factor receptor; DFS, disease free survival

## Discussion

AR is a nuclear steroid hormone receptor that is normally expressed in benign breast tissue where it is coexpressed with ER and PR in 5–30% of luminal epithelial cells [32]. AR is also expressed in metaplastic apocrine cells, a frequent component of fibrocystic change which occurs commonly in the breast. Apocrine cells show uniform and diffuse positivity for AR but do not show expression for ER or PR. AR is also reported in 60–90% of all breast cancers [35]. The biologic role of AR in breast is poorly understood. Androgen has been described as a potential tumor suppressor in ER-positive breast cancers with its anti-proliferative effect presumed to result from cross talk between steroid receptor signaling pathways [36]. However, studies investigating AR in TNBC have reported conflicting results. For example, Birell et al. noted that AR had a proliferative effect in ER and PR negative cell lines [37] which was confirmed by Garay et al. and by Doanne et al., who raised the possibility of targeting the androgen pathway [6, 38].

AR is reported in 7–75% of TNBC [13–19]. This wide range in reported incidence of AR expression in TNBC can be attributed, at least in part, to differences in immunoreactivity threshold used to define immunopositivity, in tissue fixation, in AR analysis, and/or in tumor heterogeneity. The use of tissue microarrays is another confounding factor. Currently, there are no standard or consensus guidelines for scoring AR immunoreactivity in tissue sections. We used 1% as the cutpoint to define AR+ and evaluated AR immunoreactivity in whole sections of TNBC. 1% is also the cutpoint that should be used to evaluate ER and PR positivity in breast cancers according to the ASCO/CAP guidelines [39]. A few published studies also used 1% as the cutpoint to evaluate AR immunoexpression [19, 26] while others, including a recent clinical trial showing benefit of anti-AR therapy in the metastatic setting, used a 10% as their cutpoint [27, 30, 31].

In an effort to determine the most appropriate threshold, we analyzed a cohort of 135 TNBC using several different cutpoints and found no difference in DFS of patients with cutpoints >1%. Although similar clinicopathologic correlates (occurrence in older women, apocrine morphology, and the inverse correlation with proliferation rate (Ki-67%) were noted when 25% and 1% were used as cutpoints, additional correlations with larger tumor size and increased incidence of node metastasis were observed only with 1% as the cutpoint. Therefore, 1% was considered the most appropriate cutpoint for evaluation of AR by immunohistochemistry. Adoption of 1% immunoreactivity to define AR+ TNBC would also allow the largest number of patients to benefit from targeted therapies.

Several studies have reported AR immunoexpression as a favorable prognostic factor associated with lower clinical stage, lower histologic grade, lower mitotic score, and better outcome (5-year DFS and overall survival (OS) [18–24, 40]. Although we observed a lower mitotic rate in our AR+ TNBC, the AR+TNBC in our study were larger (mean size) and had a higher incidence of axillary lymph node metastasis than the AR- tumors. Moreover, in our study there was no significant difference in mean DFS between AR+ and AR- TNBC. Our findings are similar to those reported by Pistelli et al., who observed an inverse relationship between AR expression and Ki-67% as well as a higher incidence of lymphovascular invasion, but no association with DFS or OS [30].

AR expression in the LAR molecular subtype has been shown to be 10X that in non-LAR TNBC. LAR TNBC usually express low levels of basal biomarkers and are predominantly subclassified in the non-basal subgroup [38]. Conversely, the basal-like TNBCs have low levels of AR expression. Variable levels of EGFR and CK5/6 (basal markers) positivity were noted in our AR+ cases, consistent with the expression of AR in both the LAR and basal molecular subtypes of TNBC. In our prognostic stratification, the AR+EGFR- tumors showed the best prognosis and probably represent the LAR molecular subtype, whereas, the AR-EGFR+ tumors had the worst prognosis and likely represent the basal TNBC. AR+EGFR+ tumors and AR-EGFR- tumors had an intermediate prognosis. Our prognostic groups also showed differences in proliferative rates (Ki-67%). The low-risk group had the lowest Ki-67 index, a feature that is consistent with the LAR molecular subtype. Given that chemotherapeutic agents used in the treatment of TNBC are most effective in tumors with a high proliferative rate, this group would be expected to show a poor response to chemotherapy. This may, at least in part, explain the poor response of AR+ tumors to chemotherapy, resulting in an outcome similar to that in the AR- TNBC in our study. Thus, the low-risk (AR+EGFR-) TNBC may benefit the most from antiandrogen targeted therapies. The high-risk (AR-EGFR+) TNBC had the highest proliferation rate and, therefore, might be expected to benefit the most from chemotherapy. Our findings warrant further studies for validation in larger cohorts of AR+TNBC and in controlled prospective clinical trials.

## Supporting information

S1 Fig Disease free survival of EGFR positive and EGFR negative TNBC at the threshold of 15%, for group A (study set, n = 35) at left, and group B (validation set, n = 100) at right. (TIFF)

S2 Fig AR immunoexpression in TNBC. 1–24% of neoplastic cells are positive. 20x.
(TIFF)

S3 Fig EGFR expression in AR positive TNBC. More than 15% of neoplastic cells are positive. 20x.
(TIFF)

S1 Table Disease free survival of AR positive and AR negative TNBC at different thresholds of AR. **A.** group A, study set, n = 35 and **B.** Group B, validation set, n = 100.
(DOC)
