# Supplementary figures and images for: Androgen receptor positive triple negative breast cancer: Clinicopathologic, prognostic, and predictive features
Source: PLoS One. 2018 Jun 8;13(6):e0197827. doi: 10.1371/journal.pone.0197827 (PMC5993259; doi:10.1371/journal.pone.0197827)

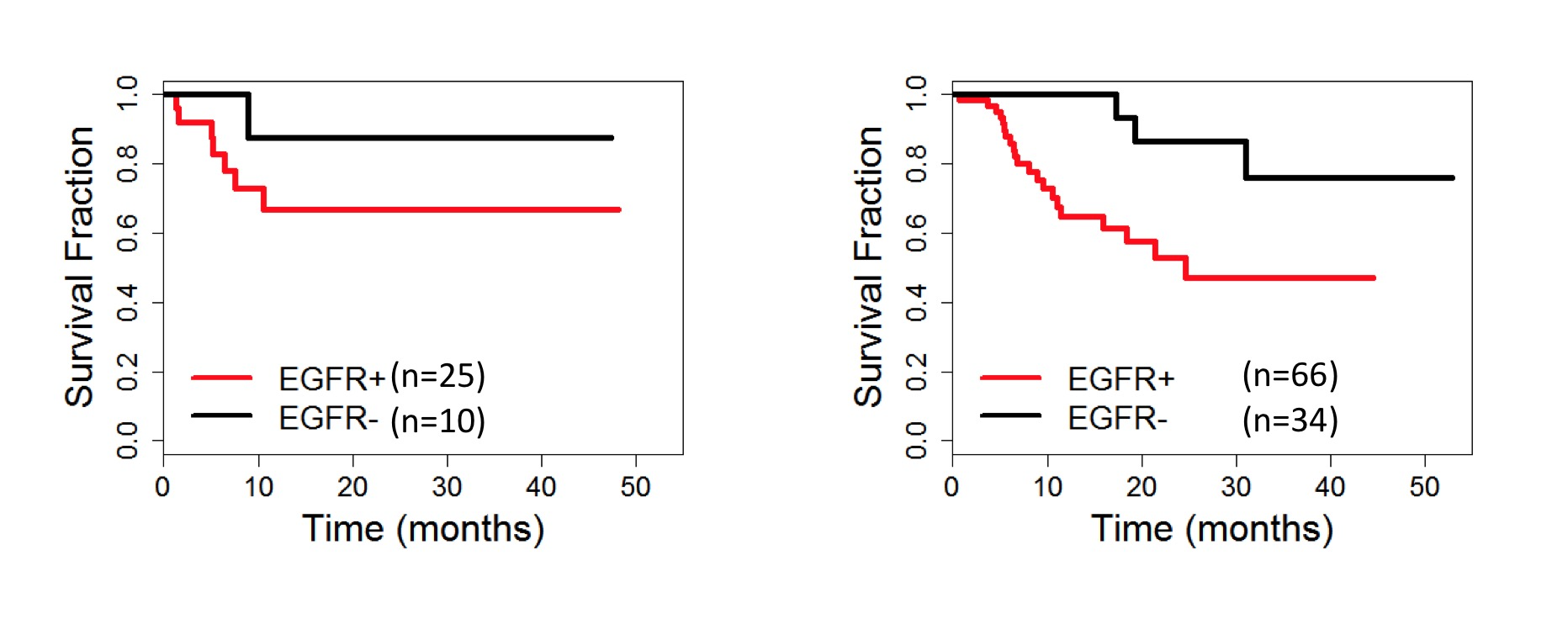

Supplement: S1 Fig — (TIFF) [file pone.0197827.s001.tiff]

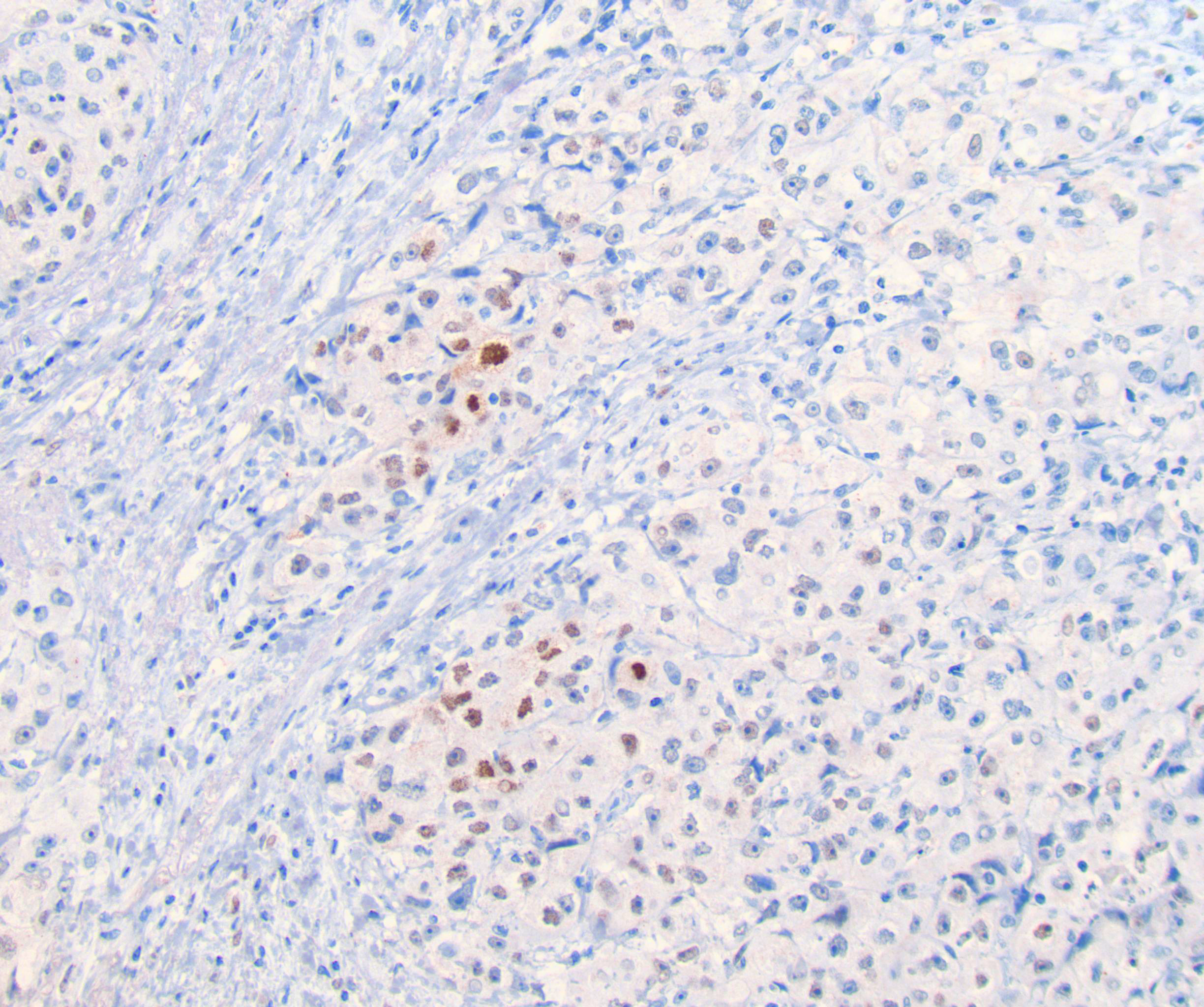

Supplement: S2 Fig — 1–24% of neoplastic cells are positive. 20x. (TIFF) [file pone.0197827.s002.tiff]

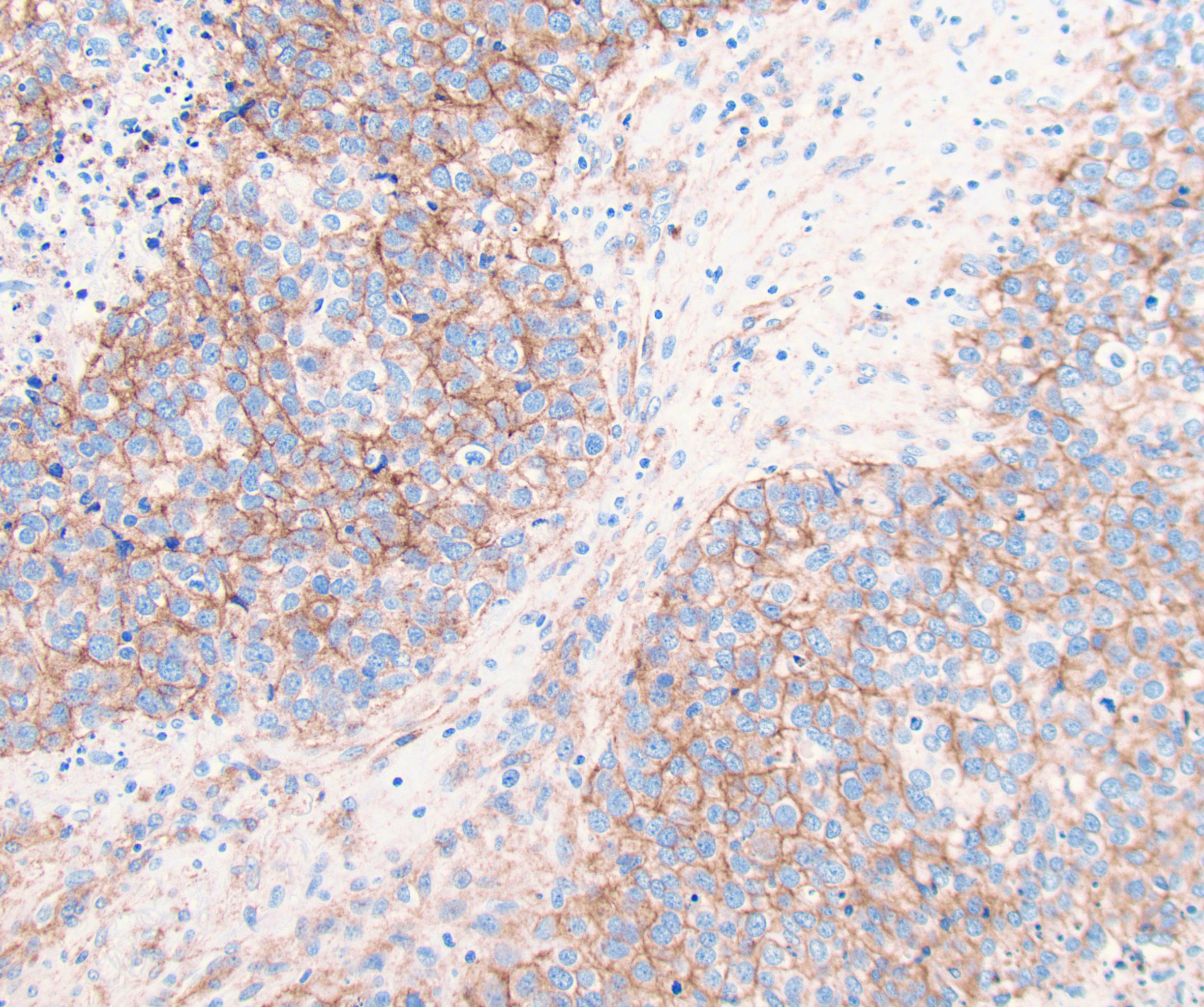

Supplement: S3 Fig — More than 15% of neoplastic cells are positive. 20x. (TIFF) [file pone.0197827.s003.tiff]
