# Supplementary material for: Androgen receptor positive triple negative breast cancer: Clinicopathologic, prognostic, and predictive features
Source: PLoS One. 2018 Jun 8;13(6):e0197827. doi: 10.1371/journal.pone.0197827 (PMC5993259; doi:10.1371/journal.pone.0197827)
Supplement: S1 Table — A. group A, study set, n = 35 and B. Group B, validation set, n = 100. (DOC) [file pone.0197827.s004.doc]

**Supplementary Table 1: Disease free survival of AR positive TNBC at different thresholds of AR.**

**A: Group A, study set,** n = 35

| **AR cutpoint values** | **AR Negative No. of Patients (%)** | **AR Positive**  **Mean DFS (Months)** | **AR Postive No. of Patients (%)** | **AR Negative**  **Mean DFS (Months)** | **Survival difference p-Value^a^** |
| --- | --- | --- | --- | --- | --- |
| ≥1 vs <1 | 14 (40%) | 35.5  +/- 4.24 | 21 (60%) | 35.4  +/- 5.53 | 0.981 |
| ≥10 vs <10 | 11 (31%) | 34.1  +/- 4.26 | 24 (69%) | 38.1  +/- 5.32 | 0.614 |
| ≥20 vs <20 | 10 (28%) | 29.2  +/- 3.29 | 25 (72%) | 31.1  +/- 4.73 | 0.782 |
| ≥25 vs <25 | 10 (28%) | 29.2  +/- 3.29 | 25 (72%) | 31.1  +/- 4.73 | 0.782 |
| ≥30 vs <30 | 9 (26%) | 28.1  +/- 3.31 | 26 (74%) | 34.5  +/- 3.91 | 0.378 |

AR, androgen receptor; TNBC, triple negative breast cancer; DFS, disease free survival.

^a^ p values were calculated using the Wilcoxon test model.

**B: Group B, validation set, n = 100**

| **AR cutpoint values** | **AR Positive No. of Patients (%)** | **AR Positive**  **Mean DFS (Months)** | **AR Negative No. of Patients (%)** | **AR Negative**  **Mean DFS (Months)** | **Survival difference p-Value^a^** |
| --- | --- | --- | --- | --- | --- |
| ≥1 vs <1 | 40 (40%) | 31.0  +/- 2.74 | 60 (60%) | 33.1  +/- 3.48 | 0.621 |
| ≥10 vs <10 | 30 (30%) | 31.1  +/- 2.44 | 70 (70%) | 30.8  +/- 3.86 | 0.994 |
| ≥20 vs <20 | 27 (27%) | 30.7  +/- 2.41 | 73(73%) | 32.3  +/- 3.85 | 0.616 |
| ≥25 vs <25 | 24 (24%) | 30.6  +/- 2.36 | 76 (76%) | 32.8  +/- 4.06 | 0.688 |
| ≥30 vs <30 | 23 (23%) | 30.2  +/- 2.35 | 77 (77%) | 34.9  +/- 3.76 | 0.452 |

AR, androgen receptor; TNBC, triple negative breast cancer; DFS, disease free survival.

^a^ p values were calculated using the Wilcoxon test model.
